# Reactive Astrocytes in Brain Metastasis

**DOI:** 10.3389/fonc.2017.00298

**Published:** 2017-12-11

**Authors:** David Wasilewski, Neibla Priego, Coral Fustero-Torre, Manuel Valiente

**Affiliations:** ^1^Brain Metastasis Group, Spanish National Cancer Research Center (CNIO), Madrid, Spain; ^2^Bioinformatics Unit, Spanish National Cancer Research Center (CNIO), Madrid, Spain

**Keywords:** brain metastasis, reactive astrocytes, metastases therapy, microenvironment heterogeneity, astrocyte signaling

## Abstract

Brain metastasis, the secondary growth of malignant cells within the central nervous system (CNS), exceeds the incidence of primary brain tumors (i.e., gliomas) by tenfold and are seemingly on the rise owing to the emergence of novel targeted therapies that are more effective in controlling extracranial disease relatively to intracranial lesions. Despite the fact that metastasis to the brain poses a unmet clinical problem, with afflicted patients carrying significant morbidity and a fatal prognosis, our knowledge as to how metastatic cells manage to adapt to the tissue environment of the CNS remains limited. Answering this question could pave the way for novel and more specific therapeutic modalities in brain metastasis by targeting the specific makeup of the brain metastatic niche. In regard to this, astrocytes have emerged as the major host cell type that cancer cells encounter and interact with during brain metastasis formation. Similarly to other CNS disorders, astrocytes become reactive and respond to the presence of cancer cells by changing their phenotype and significantly influencing the outcome of disseminated cancer cells within the CNS. Here, we summarize the current knowledge on the contribution of reactive astrocytes in brain metastasis by focusing on the signaling pathways and types of interactions that play a crucial part in the communication with cancer cells and how these could be translated into innovative therapies.

## Introduction

Brain metastasis defines the secondary tumor formation within the brain and typically results from metastases of lung cancer, breast cancer and melanoma together with other primary tumors that less frequently metastasize in the brain, such as colorectal cancer (1). We will focus on metastatic cells invading the brain parenchyma in contrast to the less frequent invasion of the leptomeninges by cancer cells, which has a very different biology derived from its location (meningeal space filled with cerebrospinal fluid) and cellular components of the microenvironment (2). Brain metastasis accounts for the major part of intracranial malignancies (3) and its incidence has been suggested to be on the rise owing to: improved imaging modalities as well as a generally lower threshold to schedule MRI imaging by physicians nowadays, extension of overall survival time of patients being treated with targeted antibody-based therapies (e.g., trastuzumab) or small molecule inhibitors (e.g., the small molecule ALK kinase inhibitor crizotinib), thus increasing likelihood for recurrence with central nervous system (CNS) lesions accounting for a main part of relapses, “sanctuary site levels” of pharmacological agents because of poor drug penetration as demonstrated for trastuzumab (4–7). Upon diagnosis of brain metastasis, affected patients suffer from significantly increased overall morbidity and mortality (1). Aside from being recognized as a serious obstacle to the care of cancer patients, only recently new insights into the molecular mechanisms accounting for metastatic spread to and growth within the brain have been made and new trials for assessing treatments in brain metastasis have been initiated to avoid traditional exclusion of this patient collective (8). Over the past decade, metastasis research with regard to the use of experimental mouse models of brain metastasis shed some light into the molecular and cellular events inherent to cancer cell dissemination and growth in the brain, which likely depends on the evolution of a series of cancer cell traits that are not necessarily required and exploited in other extracranial locations and that continue to be characterized (9–18). Though metastatic organotropism (site-specific metastasis) to different organs seems to employ some shared molecular mechanisms involved in cancer cell–host cell interactions across different tumor entities, metastasis to the brain as such is unprecedented in that the brain microenvironment harbors unique cellular and non-cellular elements and a higher degree of isolation and protection mediated by the blood–brain barrier (BBB) from both circulating molecules and cells found in the systemic circulation. Therefore, it is conceivable that cancer cells that are able to trespass the BBB and extravasate from brain capillaries face a complete different and unfamiliar tissue microenvironment subjecting cancer cells to strong selective forces (10, 19). Accordingly, cancer cells that are able to generate macrometastasis correspond to those seeds with the highest ability to integrate in such a demanding microenvironment, arguing against the BBB as the solely impediment to colonize and initiate outgrowth in the brain. The brain includes not only neurons but also glia. The glial compartment is involved in responding to any type of brain injury, such as astrocytes and microglia, the two main glial cell types together with oligodendrocytes, have been reported surround brain metastases (10, 20). Although the role of oligodendrocytes and, to a less extent, microglia has been poorly studied (21–23) in the context of brain metastasis, relatively abundant bibliography have considered brain metastasis-associated astrocytes. In this regard, recent discoveries provide compelling evidence that astrocytes, the major glial cell in the CNS, play an intricate role in brain metastasis by engaging different modes of interactions with incoming cancer cells. Although our knowledge on the crosstalk between astrocytes and cancer cells is still insufficient, recent seminal findings indicate that interactions with astrocytes occur at both early and late stages of the colonization process. Given that these interactions could provide both anti- and prometastatic stimuli to cancer cells characterizing them might aid in dissecting the molecular machinery in order to explore innovative targeted therapeutics in brain metastasis. Here, we summarize them to expose the importance of astrocytes in the biology of brain metastasis. We envision that understanding the impact of astrocytes, as one of the key host cell type in the pathogenesis of brain metastasis, may serve not only to understand the functional importance of the microenvironment in the development of this secondary tumor growth in the brain, but also to explore additional implications related to biomarkers and therapies.

## What are Reactive Astrocytes (RAs) and How have They been Studied?

Reactive astrocytes are ubiquitously present in any brain injury (24, 25). As such they have been extensively described surrounding brain tumors including brain metastasis (10, 12, 20, 26, 27). Usually they are identified by their profound alterations including the gain of a hypertrophic phenotype as well as the upregulation of the cytoskeletal intermediate filament protein glial fibrillary acidic protein (GFAP) (24, 25). However, the word *reactive* indicates a more extensive number of changes (24) to be able to face a situation in which homeostasis has been compromised. There are many stimuli that could be informative to astrocytes of such a situation and which are commonly classified as danger-associated molecular patterns (DAMPs) and pathogen-associated molecular patterns (PAMPs) (28). PAMPs are generated by microbial infections (e.g., LPS) and usually provoke a primary immune response in the CNS through microglial cells and perivascular macrophages. In contrast, the exact identity and origin of DAMPs responsible to activate the reactive program in astrocytes in the context of brain metastasis remains unknown. The fact that very limited number of cancer cells, independently of the source of the primary tumor or oncogenomic profiles, from very early stages of colonization (i.e., when lodged with the brain capillaries during the process of extravasation) (10, 20) are able to trigger this response might indicate that, at least at these initial phases, tissue injury induced by cancer cells rather than DAMPs produced by cancer cells, would be responsible for triggering the activation. Throughout cancer cell evolvement and proliferation in the CNS the stimuli influencing the reactive state in astrocytes might underlie changes. In this sense different phases related to the behavior of RAs toward insults or tissue injuries have been described encompassing an acute phase and a chronic one, which is usually referred as to glial scar (24). The acute phase is usually responsible for limiting the extension of the damage (29), however, if this cannot be achieved the response becomes chronic, which usually impairs the ability of the CNS tissue to recover from the damage completely (30, 31). Additionally, different types of brain injuries have been associated with different transcriptomic changes in RAs (32, 33), which has lead to the proposal of a dichotomy similar to the one initially applied to macrophages and microglia (34). A similar situation seems to take place in the context of brain metastasis. Early on, RAs acting as a primary host defense efficiently limit the progression of incoming metastatic cells (10), whereas later RAs have been extensively described to promote the growth of cancer cells (9, 35–38).

A significant proportion of publications considering RAs in the field of brain metastasis research are based on data generated *in vitro* exclusively, using primary mouse astrocytes or an immortalized astrocyte cell line (27, 39–43). Techniques for *in vitro* culture of astrocytes were described long time ago (44), however, recent data have demonstrated important considerations that must be taken into account. Most common protocols use early postnatal brains to obtain primary cultures of astrocytes (44). Since young and aged astrocytes could differ molecularly (45, 46) these astrocytes might not mimic those coexisting with cancer cells in the brain. Another caveat of working with astrocytes *in vitro* is that under regular culture conditions they instantly become reactive. In fact, the most widely applied method to assure the purity of the culture is to evaluate that >90% of the cells are GFAP+ (47). Since inducers of the reactive state *in vitro* likely differ from those present in secondary brain tumors, *in vitro* asytrocyte cultures used in these studies unlikely reproduce the disparity of phenotypes associated with RAs *in vivo* (48). Thus, validation of *in vitro* findings using *in vivo* approaches is an absolute requirement (a *sine qua non* condition) to generate reliable data aimed to develop potential therapeutics to target astrocytes in disease.

More advanced cultures including the addition of other cell types from the brain (10, 33), *ex vivo* brain organotypic cultures (10, 49) or brain organoids (50) are excellent platforms since they recapitulate closer the *in vivo* situation. Importantly, when applying these more sophisticated *in vitro* approaches it was found that the antimetastatic behavior of RAs, occurring during the early stages of colonization *in vivo*, was reproduced (10, 49). Alternatively, novel methodologies based on immunopanning allow avoiding the default reactive state of this cell type in culture (45). Interspecies variability and cross-species differences in cell–cell interactions need to be considered when working with non-syngeneic *in vitro* or *in vivo* systems. Differences have been reported between murine and human astrocytes regarding different aspects of their biology including the complexity of arborization, calcium response properties and transcriptomic profiles (47). Consequently findings obtained with mouse astrocytes, require validation in human samples if knowledge generated is aimed to be translated in a bench-to-bedside manner.

Up to now, studies involving RAs *in situ* are based on fixed tissue samples that evaluate GFAP+ cells (9, 10, 20, 37, 51) and none of them have reported the use of engineered astrocytes *in vivo* in the context of brain metastasis. However, as the interest in brain metastasis-associated RAs is gaining momentum and their examination will presumably be expanded toward the use of available and widely validated tools such as genetically engineered mouse models (GEMMs) that could drive reporters and/or genes of interest (29, 52, 53) in astrocytes or alternative approaches such as adeno-associated virus that target astrocytes (54) as well as *in vivo* electroporation with Star Track technology (55). Such experimental resources will need to be combined with brain metastasis models in order to determine the impact of the modifications introduced in astrocytes in the process of brain colonization by cancer cells. Spontaneous brain metastases from orthotopic injections of cancer cells (injection in the organ source of the primary tumor from which brain metastasis models were established) or GEMM that develop primary tumors are rare events and difficult to study (18, 56, 57). In contrast, models in which brain metastases are induced upon inoculation of metastatic cells in the circulation (9–12, 58–61) are compatible to study the interaction between metastatic cells and RAs during brain colonization. In these models functional experiments to dissect these interactions can be performed and analyzed using a variety of techniques such as non-invasive molecular imaging, intravital imaging and detailed histology. Whether reported differences between mice and human astrocytes (47) are relevant in the context of brain metastasis will require specific validation of experimental findings in human samples. Given the broad diversity of brain metastasis models including different tumor types, oncogenomic profiles and different species of cancer cells (human and mouse), the use of several available experimental models to confirm potential mediators of the interaction between cancer cells and astrocytes will be a good strategy to reach relevant conclusions with higher possibilities to be translated to patients (Table 1).

**Table 1:** Research goals for brain metastasis-associated reactive astrocytes.

1	Incorporate novel approaches to manipulate astrocytes *in vitro* (i.e., immunopanning) and *in vivo* (i.e., GEMM).
2	Apply unbiased genomic and proteomic analysis of reactive astrocytes (single cell level and population level) associated with brain metastasis from different primary sources (i.e., lung cancer, breast cancer, melanoma).
3	Comparison of brain metastasis-associated reactive astrocytes with those present in other brain injuries (i.e., primary brain tumors, neurodegenerative disorders, ischemia, traumatic brain injury, autoimmune disorders).
4	Identify and characterize specific subpopulations within brain metastasis-associated reactive astrocytes and evaluate their potential therapeutic implications.
5	Dissect the biology behind antimetastatic and prometastatic reactive astrocytes: Are they different subpopulations? Do they coexist in time? Could prometastatic reactive astrocytes be transformed into antimetastatic astrocytes?
6	Does systemic disease (primary tumor and extracranial metastases) influence the brain microenvironment acting on reactive astrocytes before metastases are established in the brain?
7	Do brain metastasis-associated reactive astrocytes influence systemic disease outside the brain and/or organismal homeostasis as shown in other brain disorders?
8	Could reactive astrocytes associated with brain metastasis be the source of biomarkers for early diagnosis or response to therapy?

Thus, a growing number of resources to study RAs will certainly help to understand the complexity underlying their reciprocity with cancer cells in brain metastasis.

## Astrocytes as a Source of Secreted Molecules

**Figure 1:**
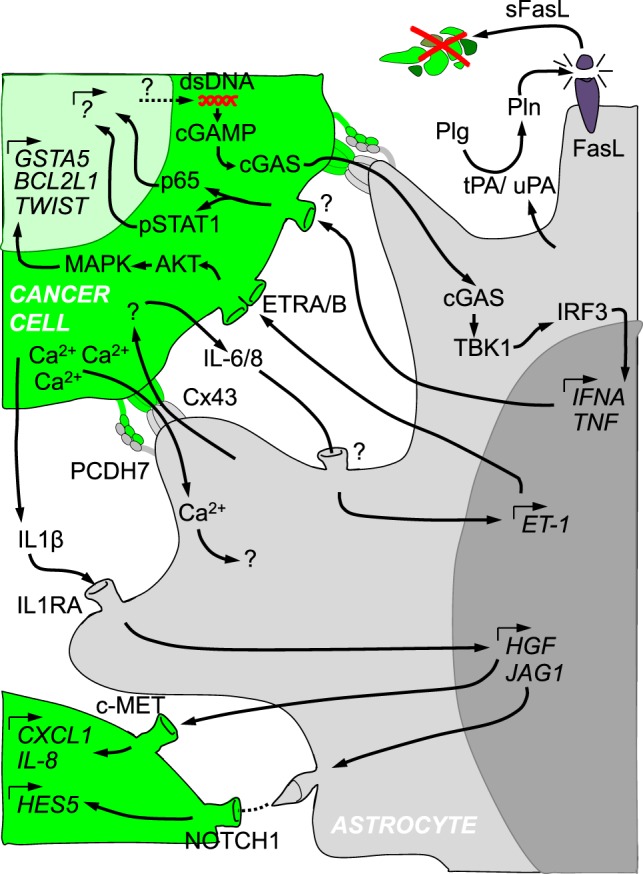
Crosstalk between cancer cells and reactive astrocytes in brain metastasis. Cancer cells (in green) and astrocytes (in gray) are depicted with several of the molecular mechanisms described in their reciprocal crosstalk. The initial ability of reactive astrocytes to kill cancer cells through the production of Plasminogen activators is later modified into a supportive niche that involves secreted molecules, gap junctions, protocadherins, Notch receptor and ligands, among other components. Such a complex interactome influences each other cell type at the gene expression level.

**Figure 2:**
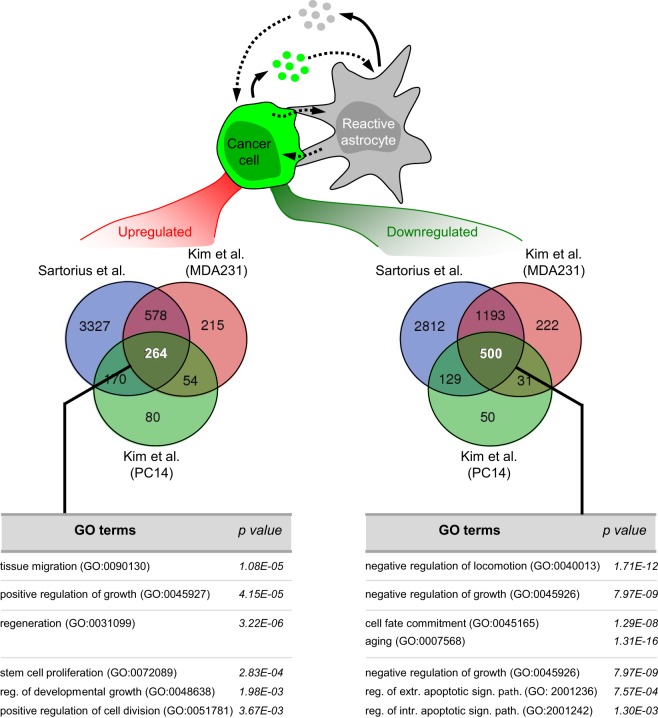
Pathway analysis on the influence of astrocytes on cancer cells *in vitro*. Bioinformatic analysis of available datasets reporting transcriptome of cancer cells upon coculture with astrocytes (27, 68) allowed us to obtain commonly 264 upregulated and 500 downregulated pathways. Some of these pathways are shown. Reg, regulation; Extr, extrinsic; Intr, intrinsic; Sign, signaling; Path, pathway.

Main findings related to reactive astrocytes in the context of brain metastasis usually include the secretory nature of this glial cell type (Table 2). Upon the first encounter with metastatic cells RAs produce plasminogen activators (PAs), including secreted tissue PA. PAs have the ability to transform plasminogen into the protease plasmin which is responsible for the elimination of many cancer cells that cross the BBB (10). Consequently, the secretory ability of RAs during the initial stages of colonization limit metastatic progression (Figure 1). However, few cancer cells produce anti-PA serpins and consequently block the antitumor program derived from RAs (10). Cancer cells with the ability to counteract the innate defense of RAs will continue colonizing the brain. Conversely, upon the development and growth of metastasis, RAs have been shown to generate a protumorigenic niche through various mechanisms involving secreted molecules. For instance, increased expression of *COX2* in brain metastatic cancer cells (MDA231BrM) has been linked to astrocyte activation and the production of CCL7 by this glial cell type (62). Astrocyte-derived CCL7 was associated with an increase in CD24low-CD44high-ESAhigh subpopulation of MDA231BrM cells. Additional paracrine cytokine signaling loops between tumor-associated astrocytes and cancer cells in breast cancer brain metastasis have been described (Table 2). Astrocytes secrete hepatocyte growth factor/scatter factor (HGF/SF) under the influence of cancer cell-derived IL1β (Figure 1). Targeting this mutual c-Met-HGF crosstalk between cancer cells and tumor-associated astrocytes by using the BBB-permeable compound Pterostilbene, a resveratrol analog, diminished the stem-like cell phenotype dependent upon this feed-forward signaling loop both *in vitro* and *in vivo* (63). Another growth factor, namely BDNF, was suggested to be linked to the interplay between astrocytes and breast cancer cells in brain metastasis. Astrocyte-derived BDNF can favor cancer cell proliferation by engaging heterodimerization of both Her2/NEU and TrkB receptors *in vitro*. Importantly, inhibition of this crosstalk by means of knocking down TrkB in cancer cells abrogated brain metastasis *in vivo* (36). In line with these studies, a previous one reported that brain metastatic cancer cells significantly up regulate IL-1β, which again seems to be embedded in a mutual signaling loop between cancer cells and astrocytes. This was associated with a cancer cell-mediated activation of astrocytes, reflected by a heightened expression and production of astrocytic Jagged1 in a NFκβ-dependent manner (64) (Figure 1). Accordingly, the resulting paracrine interaction between Jagged1 + astrocytes and cancer cells was able to increase the stem-like phenotype in cancer cells *via* the Notch-Hes5 pathway (64). In the context of melanoma-to-brain metastasis evidences exist arguing about the ability of cancer cells to reprogramme astrocytes (understood as the induction of transcriptional modifications providing prometastatic functions). Cancer cells were able to induce the production of IL-23 in RAs. This proinflammatory cytokine was shown to be of importance in the up-regulation of cancer cell-derived MMP2, which in turn mediates invasiveness of brain metastatic melanoma cells *in vitro*. Blocking this paracrine interaction either by pharmacological inhibition of IL-23 or by knocking down cancer cell MMP2 resulted in inhibition of melanoma invasion *in vitro* (65). A recent study further supports a potential role of MMP2 in breast-to-brain metastasis, as it was found to belong to 5-gene expression signature (together with *CXCL12, MMP11, VCAM1, MME*) discriminating between primary breast cancer and breast cancer brain metastases (66). To sum up, it seems to be evident that cancer cells get assistance originating from astrocytes after hijacking those and/or transforming them into passive bystanders sustaining migration and growth as well as tumor-initiating capabilities of cancer cells (Figure 2).

**Table 2:** Secreted molecules by brain metastasis-associated reactive astrocytes.

RA secreted molecules	Phenotype in cancer cells	Cancer type	Reference
ET-1	Induction of cancer cell growth and stemness through activation of MAPK and AKT	Breast and lung cancer	Kim et al. (35)
IL-23	Increase invassiveness by inducing MMP2	Melanoma	Klein et al. (65)
HGF/SCF	Induction of proangiogenic cytokines by activating c-Met	Breast cancer	Xing et al. (63).
BDNF	Induction of cancer cell growth through activation of TrKB-HER2	Breast cancer	Choy et al. (36)
CCL7	Induction of tumor initiating potential in cancer cells	Breast cancer	Wu et al. (62)
MMP2/MMP9	Increase invassiveness of cancer cells	Breast cancer	Wang et al. (67)
miR-19a	Induction of cancer cell growth by targeting PTEN	Breast cancer and melanoma	Zhang et al. (37)
Hyaluronic acid	Induction of cancer cell growth and stemness through activation of MAPK and AKT	Lung cancer	Stevens et al. (38)
IFNα/TNFα	Induction of cancer cell growth and chemoresistance by STAT1 and NFκB	Breast and lung cancer	Chen et al. (9)
PA-Plasmin-FasL	Decrease the viability of non-brain-adapted cancer cells	Breast and lung cancer	Valiente et al. (10)

## Cancer Cell–Astrocyte Interactions through Gap Junctions

Astrocytes form an interconnected network that allows signal transduction in a coordinated manner (69). Key players in this communication are gap junctions. Gap junctions are composed of connexins (Cxs). Out of the 21 reported Cxs, Cx43 (also referred to as GJA1) and Cx30 (GJB6) are the most abundant in the adult brain (69). In physiology, astrocytic gap junctions are required for proper neuronal activity, synaptic transmission, energy supply and control of blood flow (69). When astrocytes become reactive their functional and spatial domains can be altered which might also modify their connectivity (70). Interestingly, initially *in vitro* (27, 40) and later *in vivo* (9), brain metastatic cells have been shown to be able to establish gap junctions with RAs. Why do brain metastatic cells have developed this ability? The Fidler lab addressed whether besides secreted molecules from RAs additional interactions involving physical contact with cancer cells could benefit them (27, 40). Their rationale was based on the conspicuous proximity between some cancer cells from established metastasis and RAs *in vivo* (41). Their series of articles probed the physical interaction between them, its dependency on Cxs and the benefit it provided to cancer cells (27, 40). Through this cell–cell interaction, astrocytes induced the expression of 205 genes in different cancer cell lines from breast and lung cancer (27). Although this gene program was expected to be Cx-dependent, the authors did not clarify this aspect since replicas including carbenoxolone, a gap junction inhibitor, or shRNA against Cx43 were not part of the transcriptomic profile. Hence, the resulting assumptions drawn from this gene list warrant further evaluation in respect to the dependency on Cx43. This concern was further enlarged given their findings by which calcein (a gap junction permeable molecular dye) is also transferred between fibroblasts and cancer cells although, in contrast to astrocytes, this cell type did not potentiate cancer cell survival in the presence of chemotherapies (27). Consequently, these results seem to be inconclusive regarding the dependence of the gene program induced in cancer cells by the influence of astrocytes. However, this point was partially clarified later when IL-6 and IL-8 production from cancer cells was shown to be dependent on the establishment of gap junctions with astrocytes (35). These cytokines influence both cancer cells and astrocytes, by inducing the expression of both endothelin receptors (ETAR and ETBR) on cancer cells and endothelin ligand (ET-1) on astrocytes (35) (Figure 1). Few of the initially deregulated genes upon cancer cell–astrocyte interaction were probed to be dependent on ET-1 (35). A number of these genes, including mesenchymal genes (*TWIST1)*, inducers of resistance to stress (*GSTA5)*, and antiapoptotic genes (*BCL2L1)*, were validated in human brain metastasis (35). Based on these findings, they provided evidence that chemotherapeutic drugs including paclitaxel, 5-fluorouracil (5-FU), and cisplatin killed less cancer cells than when the gap junction inhibitor carbenoxolone was added to cocultures or Cx43 was knocked down in astrocytes (27, 40). Yet, there were neither validations of the roles of these genes *in vivo* nor a molecular explanation on how gap junctions were established in the first place between the different cell types. Further, it remains to be seen whether the genes identified are main players in the brain metastasis phenotype. In contrast, the molecular mechanisms underlying the establishment of gap junctions between cancer cells and RAs has been recently reported (9). A gene list initially reported as commonly deregulated in brain tropic cancer cells from lung and breast cancer, included the protocadherin 7 (*PCDH7*) (10). Besides brain metastatic cells, *PCDH7* was also expressed in RAs *in vitro* and *in vivo* but not in other brain cell types (9). When *PCDH7* was downregulated from brain tropic cells, gap junction mediated transfer of calcein to RAs was severely impaired. Detailed analysis of the PCDH7-dependency of Cx43 mediated gap junction communication probed that the protocadherin is required to establish gap junctions between cancer cells and astrocytes (Figure 1). Once the gap junction channel connects both cell types, they exchange at least two types of molecules that have been described in brain metastasis: the ion Ca^2+^ and the secondary messenger cGAMP. Calcium is usually exchanged between astrocytes within the neural network to synchronize their activity and coordinate their responses under homeostatic conditions (69). Cancer cells from multiple brain metastastasis models were shown to co-opt gap junction communication with astrocytes to reduce their excessive calcium load (Figure 1). Excessive amounts of calcium could be detrimental for cancer cells since it is a known trigger of DNA damage and inducer of apoptosis (71). As a consequence, a decrease in the intracellular concentration of calcium seems to be a requirement to maintain an aggressive brain colonization pattern with marked resistance to chemotherapy. Intriguingly, use of gap junctions by cancer cells includes mechanisms reminiscent to antiviral cellular responses as shown previously (72, 73). Genomic instability is a frequent finding in advanced metastatic cancer (74) and as such has been reported in brain metastasis (75, 76). Although beneficial for cancer cells by boosting the generation of genetic variants that might be better fitted to colonize the brain, genomic instability also generates toxic byproducts such a double-stranded DNA (dsDNA) (77). Cytosolic dsDNA is sensed by cGAS which upon activation generates the second messenger cGAMP, driving an interferon response upon activation of STING (78). Transfer of cGAMP from cells infected with viruses to surrounding cells through Cx43 dependent gap junctions was described as a mechanism to prevent viral expansion (79). Hence, brain metastatic cells have managed to co-opt and utilize this ancient molecular mechanisms for their own benefit. Initiated by the transfer of the second messenger to RAs, cGAMP activates STING at the endoplasmic reticulum, which leads to TBK1 mediated phosphorylation of IRF3 as described in other cellular contexts (78). Thereby, phosphorylated IRF3 enters the nuclei where it induces the expression and secretion of TNFα and INFα. These two cytokines in turn can activate NFκβ and STAT1 in brain metastatic cells, which contributes to an increase in their proliferative potential and resistance to chemotherapeutic stress (9) (Figure 1). Interestingly, dsDNA is abundant in exosomes (80). Given the reported transfer of dsDNA between cells through exosomes in which the presence of Cx43 facilitates the entry into the recipient cell (81), additional mechanisms, which do not require juxtacrine, direct cell–cell contact, might also play a role *in vivo*.

## Influence of Astrocytes on Non-Cancer Cells

As delineated above, astrocytes, as the most abundant cell type confined to the CNS, will statistically (by means of localization) account for a majority of the interactions that brain-homing clones of cancer cells will be exposed to during early but also late stages of brain metastasis. However, other cell types of the brain metastasis environment such as endothelial cells, pericytes and resident microglia as well as incoming myeloid cells (i.e., macrophages) have been shown to interact and respond to the presence cancer cells (10, 17, 20, 22, 43, 58, 82, 83). Hence, astrocytes might also influence not only cancer cells but also other adjacent cell types in brain metastasis in a direct or indirect fashion. For example, astrocytes transfer exosome-enpacked microRNA-19a (miR-19a) to cancer cells. miR-19a silences the major tumor suppressor phosphatase and tensin homolog deleted on chromosome 10 (PTEN) in cancer cells. As a result of this interaction a more favorable adaptation of cancer cells to the new tissue environment is achieved by increasing their growth rate but also by inducing the secretion of the chemokine (C-C motif) ligand 2 (CCL2). Cancer cell secreted CCL2 participates in the generation of a protumorigenic niche by inducing an influx of brain metastasis-promoting Iba1+/CCR2+ myeloid cells. Importantly, higher CCL2 scores as determined by immunohistochemistry were more frequently seen in brain metastatic tissue than in matched primary tumor tissue and additionally CCL2 expression correlated with PTEN loss in brain metastatic tissue (37). These results were corroborated by a more recent study (43). Although insights into astrocyte-mediated influence on other cell types in the context of brain metastasis is ill-defined, recent insights into phenotypic and genotypic signatures of astrocytes in other neurological diseases and other fields of neuroscience may aid in elucidating these potential implications in regard to brain metastasis (33). Emerging evidence has also reported the influence of RAs beyond the brain (84). Secretion of extracellular vesicles by RAs, including exosomes, could reach the systemic circulation in experimental models of inflammatory brain damage. Astrocyte-derived extracellular vesicles gain access to different organs (liver, lungs and spleen) where they induce an acute cytokine response characterized by the secretion of IL-17, IL-1β, IL-6, TNF-α, and CCL2. This acute cytokine response leads to the mobilization of Ly6b+ leukocytes that will infiltrate the brain to resolve the damage (84). Whether a similar mechanism is occurring in brain metastasis remains to be addressed. Thus, future studies in brain metastasis should consider not only the local influence of RAs but also their potential contribution to other symptoms which might contribute to the deterioration of patient health state during brain metastasis (Table 1).

In contrast to RAs in other disease conditions such as stroke or traumatic brain injury, brain metastasis is a continuously progressing insult (i.e., growth and evolution of cancer cells). In this given context astrocytes are unable to resolve the insult and over time cancer cells hijack some of their functions and prompting to astrocytes to work for their own benefit. Thus, astrocytes convert to a dubious fellow companion to cancer cells aiding them in remodeling their new habitat, potentially influencing the behavior of other CNS cell types.

## Astrocyte Heterogeneity in Brain Metastasis

The brain is highly complex in respect to its cellular (and acellular) composition. The main cell type, the neuron, can be classified in two main classes (excitatory and inhibitory) and within them multiple subclasses are required to maintain the fine-tuning and wiring of neural circuits (85, 86). This complexity has remained exclusive to the neuronal compartment. However, evidence as to heterogeneity in non-neuronal components is steadily increasing. Recent findings have probed that subtypes of microglia reside in specific locations in the brain (87) which might be linked to subpopulations that emerge in and drive brain disorders of experimental models and humans (88). Astrocyte heterogeneity is of emerging interest given the potentially important implications in homeostasis (89–92) and disease (93–96). In brain metastasis in particular, a good body of evidence points toward opposite behaviors of astrocytes, which seem to be dependent on the disease stage. Initially, astrocytes act as a innate host defense system limiting the progression of the disease (10), while later on astrocytes favor it (9). Whether they belong to different subtypes of astrocytes or whether a consequence of the influence of cancer cells on them remains an issue of dispute. In view of the findings reported under homeostatic conditions and other CNS disorders, astrocytes are likely to include different subpopulations (48). Although heterogeneity in RAs associated with brain metastasis has not been formally probed, there are published observations that might be indicative of this possibility. During the colonization of the brain RAs surround brain metastatic cells (9, 10, 12, 20, 26). Besides GFAP other markers identifying this cell type have been reported in this glial cell type. However, these markers did not fully colocalize with each other, so that many GFAP+ RAs were negative for them, as in the case for Nestin. Nestin labels neural stem cells (97). The finding that Nestin is only present in a subset of RAs associated with brain metastasis (20) could suggest that heterogeneity among brain metastasis-associated astrocytes might have deeper implications at the functional level. In one study Xing et al. reported that Jagged1+ RAs were actively inducing Notch activity in brain metastatic cells (64). Again, the Jagged1 colocalization with GFAP was only partial (64), suggesting that within the population of RAs there could be also a Jagged1-subset as well. The same applies to endothelin receptor, which has been shown to be present in a heterogeneous pattern among RAs in the context of brain metastasis (98). Interestingly, endothelin receptor and Notch have been reported in reactive astrocytes in other brain injuries (95, 99). A more unambiguous example of the presence of RAs subpopulations associated with brain metastasis corresponds to the identification of p751-PDGFRβ+ astrocytes (100). Phosphorylation of Tyr751 was used to label this subpopulation of RAs associated with brain metastasis, preferentially located close to capillaries. This finding was expanded to human brain metastasis with breast and lung cancer. The inhibitor pazopanib, a multityrosine kinase inhibitor, including PDGFRβ, was used to evaluate the functional implications of this subpopulation. Pazopanib used *in vivo* in experimental brain metastasis models significantly prevented their development. Yet, given the unspecific inhibitory nature of this inhibitor and the previous report showing that another pazopanib target present in cancer cells was required for brain metastasis, makes it difficult to conclude about the potential involvement of p751-PDGFRβ+ RAs in brain metastasis. Authors probed that the phosphorylation of the PDGFRβ receptor in astrocytes was induced upon coculture with brain metastatic cancer cells, indicating that PDGFRβ+ RAs might represent a brain metastasis-specific subpopulation (Table 1).

Consequently, exploiting the molecular characterization of brain metastasis-associated RAs is an emerging area of research that will facilitate the understanding of their biology and which could also offer innovative ways to target this particular condition.

## Genomics and Signaling Pathways in Brain Metastasis-Associated Astrocytes

In contrast to existing examples in cancer cells (9, 40, 68) (Figure 2), there are no genomic data regarding RAs associated with brain metastasis. Instead, several publications have reported specific signaling pathways to be involved in the crosstalk (Figure 1). A reactive astrocytic phenotype observed in a melanoma brain metastasis model (51) was linked to an earlier proposed gene signature for RAs in stroke or LPS treatment (32). Although the authors did not undertake an unbiased astrocyte-specific profiling in their experiments gliosis-related genes such as *Gfap, Cxcl10, Lcn-2, Serpina3n, Serpine1*, and *Timp-1* were significantly upregulated in the group of mice in which melanoma cells were coinjected together with astrocytes as compared to melanoma cells injected alone (51). Besides these gene expression changes additional signaling pathways have been reported (Figure 1) including Cx43/cGAS/TBK1/IRF3/IFNα.TNF (9), IL-6.IL-8/ET-1 (35), IL1B/IL1RA/HGF.JAG1 (63, 64). Increasing numbers of genomic studies on astrocytes are being performed in other neurological disorders (32, 52, 90) which will be an extraordinary repository for comparative analyses between different brain disorders to interrogate common and different aspects of the underlying biology of RAs in different scenarios as well as to evaluate the possibility to apply drug repurposing (Table 1).

## Astrocyte-Based Therapies

Although limited in number, some studies have tested the impact of targeting certain aspects of RAs associated with brain metastasis. Since these therapeutic efforts are aimed to block prometastatic components of the microenvironment, they have been applied to advanced stages of the disease. All of these preclinical studies have shown great potential thus opening the possibility of treating brain metastasis by targeting the microenvironment (9, 98, 100). In principle, these innovative therapies might be applied to a broader number of patients, given that all brain metastases harbour RAs associated independently of the source of the primary tumor. Such therapies might also involve less secondary effects, given that the target will not be attributed to normal brain tissue.

### Macitentan

Macitentan is a FDA-approved BBB permeable inhibitor targeting endothelin receptor A and B (101) that is being used for treatment of pulmonary arterial hypertension. Macitentan has been repurposed to evaluate its potential effect in primary and secondary experimental brain tumor models (98, 102). Treatment of established experimental brain metastasis from lung (PC-14) and breast cancer (MDA231) in the preventive (micrometastasis) and interventional (macrometastasis) settings dramatically reduced brain metastasis and increased survival in mice, but only when combined with chemotherapy (Paclitaxel). Since these receptors are also present in cancer cells and endothelial cells, the therapeutic benefit cannot be assigned to targeting RAs alone. Nevertheless, given the contribution of astrocytes to endothelin receptor signaling (35) a part of it might be derived by the inhibitory effect in astrocytes. Although Macitentan alone induced a massive reduction in pAKT and pMAPK, this did not translate into a detectable phenotype with non-invasive bioluminescence monitoring. However, combination with Paclitaxel dramatically decreased the number of tumor-associated vessels, limiting the access of nutrients to cancer cells, which suffer from massive induction in cleaved caspase 3. Interestingly, initially described genes upregulated in cancer cells upon coculture with astrocytes (*BCL2L1, GSTA5*, and *TWIST1*) (27) were downregulated by Macitentan alone.

Given the finding of a similar phenotype in glioma models, a key contribution of the endothelin axis in brain tumors seems to be probable. The clinical trial initiated in recurrent glioma based on these findings (NCT01499251) was concluded recently and results should be publicly available soon. If positive results being reported, this therapeutic effort should be extended to brain metastasis patients.

### Gap Junction Inhibitors

Gap junction intercellular communication (GJIC) is more and more seen as a potential target in different disease conditions such as different heart pathologies, seizures and cancer (103). There is a good amount of *in vitro* studies dedicated toward the characterization of pharmacological modulators of GJIC either *via* acute uncoupling or enhancement of signaling or their influence on gene expression, biosynthesis and turnover (103). Yet, until recently there have been only a few studies published on the potential usefulness of inhibition of GJIC in the setting of brain metastasis (27, 40). Initial work probed the protective effect of astrocytes against different chemotherapeutic drugs used in the clinic (paclitaxel, cisplatin, and 5-FU) on different melanoma cell lines *in vitro* (40). Inhibition of GJC channels pharmacologically by using the pan-Cx inhibitor carbenoloxone (CBX) or genetically by knocking down gap junctions in astrocytes during coculturing of cancer cells with astrocytes was able to render cancer cells chemosensitive (40). The therapeutic value of targeting gap junctions in experimental brain metastasis models was recently reported (9). Instead of CBX the authors used two drugs for this purpose: the anti-inflammatory compound meclofenamate, which was previously shown to inhibit Cx43 gap junction gating (104), and the benzopyrane derivative tonabersat, which was previously shown to have specific activity for binding to astrocytes and inhibit gap-junction-mediated processes (105–107). Both were used in brain-related disorders before (107, 108) and are FDA approved. The use of either meclofenamate or tonabersat in breast or lung brain metastasis from human or mouse cancer cells induced a significant decrease in brain tumor burden even after metastatic cells have seed and grew in this organ (9). Interestingly, given the brain specific mechanism targeted, none of the drugs show any effect when applied to orthotopic injections in the breast or in the lung (9). Based on these results an ongoing clinical trial (NCT02429570) has been launched to apply meclofenamate to recurring or progressing brain metastasis from multiple primary tumors.

### Pazopanib

This orally bioavailable multiple tyrosine kinase inhibitor targeting VEGFR1–3, PDGFRα-β, c-kit, and B-Raf was initially found to target cancer cells in experimental HER2+ brain metastasis models (109). Later it was found that it also decreases tyrosine phosphorylation of PDGFRβ in RAs present in lung and breast cancer brain metastasis, as well as from astrocyte primary cultures obtained from craniotomies of five patients with brain metastases. As with Macitentan, the specific contribution of inhibition of astrocyte PDGFRβ receptor is not known. Thus, in order to conclude about the contribution of this therapy, more specific genetic strategies targeting PDGFRβ in RAs are necessary. However, the ability of pazopanib to decrease the phosphorylation of the PDGFRβ receptor in astrocytes was correlated with reduced proliferative capacity of an immortalized astrocyte cell line (100). *In vivo*, targeting of Tyr phosphorylation of PDGFRβ did not decrease the GFAP population of RAs suggesting that pazopanib targets PDGFRβ+ RAs without killing them.

### Compound E

As brain metastatic cancer cells co-opt physiological pathways to adapt to and to thrive within the CNS, the same applies to the acquisition of a stemness phenotype (64). RAs are involved in the regulation of the cancer stem-like cell phenotype in a Notch-dependent manner (64). Subsequently, Compound E, a gamma secretase inhibitor, has shown to impair the interaction between the astrocytic Jagged1 and cancer cells expressing Notch (64). When Compound E was administered to mice injected with a triple negative breast cancer model metastatic to the brain (MDA231-BrM) a significant decrease in the growth of cancer cells was observed (64).

### Lenalidomide

Currently, no predictive biomarkers are clinically available to help in identifying those cancer patients which likely will experience brain metastasis during the course of their disease. Ongoing efforts to identify brain metastasis biomarkers found FN14 as a gene differentially expressed in brain metastasis as compared to primary tumors (110). This finding was later validated in a multicenter study involving 318 breast cancer patients, in which 138 developed brain metastases (13). Intriguingly, the presence of FN14, a TNFR family receptor member, in primary breast tumors was associated with a 5.24-fold increase in brain metastasis incidence (13). FN14 ligands include astrocyte growth factors TWEAK and TNF-alpha, where the former is mainly produced by astrocytes and microglial cells in the CNS (13, 111). Tackling FN14/TWEAK axis by using the thalidomide derivative lenalidomide (LND) impaired brain metastasis presumably through an effect on RA reactivity (13). Based on its anti-inflammatory properties demonstrated in other CNS conditions such as multiple sclerosis and the relative success of lenalidomide used in the framework of a first line regimen for multiple myeloma, upcoming studies should verify the reported beneficial effect in targeting brain metastasis, possibly in combination with other first line drugs.

## Discussion

Whilst brain metastasis remains a major threat to cancer patients, its annual incidence being on the rise, extracranial disease is becoming targeted more and more efficiently owing to the advent of molecular therapeutics as exemplified most recently by immune checkpoint inhibitors (112). New insights deriving from *in vitro* and *in vivo* preclinical models of brain metastasis have enabled researchers to have a more precise picture of the biology of coevolution of brain colonizing cancer cells and their surrounding microenvironment, *per se* offering ways for therapeutic exploitation. By analogy with insights derived from the field of neuroinflammation and neurodegeneration, where RAs are increasingly seen as main disease elements by means of modulating neurotoxic effects *via* non-cell autonomous mechanisms, more in-depth and comprehensive characterization of the RA phenotype on a genomic or proteomic scale in brain metastasis [employing techniques such as population or single cell RNA-seq, TRAP, or MS-based proteome analysis (32, 33, 52, 90)] might soon become a reality and enable researchers to validate potential candidates in brain metastasis models. It would be interesting to determine whether there is a difference, and if yes to what extent RAs found in brain metastasis distinguish from their counterparts seen in other disease conditions (32, 33, 52). Additionally, insights into the role of specific astrocyte subpopulations, their evolution during disease progression as well as their manipulation would provide a valuable means of targeting astrocyte-cancer cell interactions. Importantly, one must ask whether there is any specific therapeutic window as to which time point during brain metastasis might represent the most effective way of modulating and targeting this vicious crosstalk or even promote the antimetastatic behavior of RAs. Taken together, insights gained from recent research have undoubtedly put astrocytes in brain metastasis into perspective turning them from passive bystanders to active key players in brain metastasis.

In addition to that, new avenues of research will allow studying RAs in brain metastasis on a more systemic scale, an aspect which is believed to be underestimated in the context of gliosis in other CNS diseases such as infectious diseases, where proinflammatory molecules released from peripheral tissue sites of infection likely influence far-distant RAs (28). Interestingly, emerging studies have proposed the influence of the primary tumor on the brain environment facilitating the colonization of cancer cells (113, 114). The systemic influence of RAs in models of neuroinflammation has been also reported (84, 115). Given the strong response of RAs to brain metastasis during the course of brain colonization, it is temping to speculate that the identification of secreted molecules might represent putative biomarkers of early diagnosis or response to therapy, even more if specific prometastatic subpopulations of RAs could be identified to be targeted.

In sum, RAs are largely involved in reciprocal interactions with metastatic cells and govern distinct cancer cell phenotypic features required during the process of colonization such as invasive capacity, survival and stemness. The focus of future studies will likely shift toward the specific makeup of the brain microenvironment appreciating its complexity and heterogeneity as well as its role to serve as a putative future therapeutic target in combating brain metastasis more efficiently and successfully (Table 1).

## Author Contributions

MV, NP, and DW conceptualized the work and wrote the manuscript. CF-T performed the bioinformatic analysis.

## Conflict of Interest Statement

The authors declare that the research was conducted in the absence of any commercial or financial relationships that could be construed as a potential conflict of interest.
